# Correlation of genotype and phenotype in 32 patients with hereditary hemochromatosis in China

**DOI:** 10.1186/s13023-021-02020-y

**Published:** 2021-09-28

**Authors:** Liyan Wu, Wei Zhang, Yanmeng Li, Donghu Zhou, Bei Zhang, Anjian Xu, Zhen Wu, Lina Wu, Shuxiang Li, Xiaoming Wang, Xinyan Zhao, Qianyi Wang, Min Li, Yu Wang, Hong You, Jian Huang, Xiaojuan Ou, Jidong Jia

**Affiliations:** 1 Beijing Key Laboratory of Translational Medicine On Liver Cirrhosis , Liver Research Center, Beijing Friendship Hospital, Capital Medical University, 95 Yong-An Road, Beijing, 100050 China; 2 National Clinical Research Center for Digestive Diseases, Beijing, 100050 China; 3 Liver Research Center, Experimental Center, Beijing Friendship Hospital, Capital Medical University, 95 Yong-An Road, Beijing, 100050 China; 4 Department of Clinical Epidemiology and EBM, National Clinical Research Center for Digestive Diseases, Beijing Friendship Hospital, Capital Medical University, Beijing, 100050 China

**Keywords:** Hereditary haemochromatosis (HH), Non-*HFE*, Genotype, Genotype–phenotype

## Abstract

**Background:**

Hereditary hemochromatosis (HH) is widely recognized and clinical manifestations of hemochromatosis-related (*HFE*-related) HH is well studied in European populations. Less is known about the clinical and laboratory characteristics of non-*HFE* related HH in Asian population. We aimed to explore the relationship between genotype and clinical phenotype in Chinese patients with non-*HFE* related hereditary hemochromatosis.

**Methods:**

Peripheral blood samples and clinical data of patients with primary iron overload were collected from the China Registry of Genetic/Metabolic Liver Diseases. Sanger sequencing was performed in cases with primary iron overload, for 5 known HH related genes (*HFE*, *HJV*, *HAMP*, *TFR2* and *SLC40A1)* and 2 novel iron homeostasis-related genes (*DENND3* and *SUGP2)*. The correlation of genotype and clinical phenotype in these patients was analyzed.

**Results:**

Of the 32 patients with primary iron overload (23 were males and 9 were females), non-*HFE* variants were detected in 31 (31/32, 97%), including 8 pathogenic variants in *HJV*, 7 pathogenic variants in *SLC40A1*, 8 likely pathogenic variants in *SUGP2* and 5 likely pathogenic variants in *DENND3* cases. Among these 31 cases, 4 cases harbored homozygous variants, 2 cases harbored homozygous + heterozygous variants, 19 cases harbored heterozygous or combined heterozygous variants, and 6 cases harbored no any damaging variants. None of investigated cases carried damaging *HAMP* and *TFR2* variants were found. 8 cases were classified as type 2A HH and 6 cases as type 4 HH, 10 cases as non-classical genotype, and 6 cases had no pathogenic variants from 31 cases. During the statistical analysis, we excluded one case (*SLC40A1* IVS3 + 10delGTT + *SUGP2* p. R639Q(homo)) with difficulty in grouping due to combined damaging variants. Cases with type 2A HH have an earlier age at diagnosis (*p* = 0.007). The iron index of cases in type 2A HH and type 4 HH was higher than that in other groups (*p* = 0.01). Arthropathy was relatively rare in all groups. None of cases with type 2A HH developed cirrhosis. Cirrhosis and diabetes are more prevalent in type 4 HH. The incidence of cirrhosis (*p* = 0.011), cardiac involvement (*p* = 0.042), diabetes (*p* = 0.035) and hypogonadism (*p* = 0.020) was statistically significant in the four groups. However, due to the limited sample size, the pairwise comparison showed no significant difference.

**Conclusions:**

This is the first comprehensive analysis about the gene variant spectrum and phenotypic aspects of non-*HFE* HH in China. The results will be useful to the identification, diagnosis and management of HH in China.

## Introduction

Hereditary haemochromatosis (HH) is an iron-storage disease, caused by mutations in genes involved of the regulation of iron homeostasis, resulting in excessive absorption and toxic accumulation of iron in the liver, pancreas, skin, heart, joints, and anterior pituitary gland [1]. In untreated individuals, iron overload can lead to liver fibrosis/cirrhosis, diabetes, skin pigmentation, heart disease, bone and joint disease, and hypogonadism. Hereditary hemochromatosis is associated with malignancies, particularly hepatocellular carcinoma [2]. Early recognition, diagnosis and treatment for hemochromatosis can reduce iron deposition and prevent disease progression [3, 4].

There are 4 main types of HH that have been categorized based on which proteins involved in iron homeostasis are affected [5]. Type 1 HH is the most common form of HH in Caucasian populations, which is caused by homozygous p. C282Y or compound heterozygous p.C282Y/H63D mutations in *HFE* gene [6]. Type 2A, type 2B, type 3, and type 4 are associated with pathogenic defects in the hemojuvelin (*HJV*), hepcidin (*HAMP*), transferrin receptor 2 (*TFR2*) and ferroportin (*SLC40A1*) genes, respectively [7]. Mutations in the *HFE*, *HJV*, *HAMP* and *TFR2* genes result in an autosomal recessive form of HH, whereas mutation in the *SLC40A1* gene results in an autosomal dominant form of HH [7].

The majority of HH cases are related to non-*HFE* genes in Asian countries [8, 9]. In addition, some novel gene variants related to the regulation of iron homeostasis have been identified in Chinese HH patients. In our previous study, *SUGP2* p. R639Q, *BMP4* p. R269Q, and *DENND3* p. L708V were first identified in HH patients [10].

However, less is known about the clinical features and genetic correlation of non-*HFE* HH in Asian populations. Therefore, in the present study, we investigated the genetic characteristics and relationship between genotype and phenotype of non-*HFE* HH in a cohort of patients with HH in China.

## Methods

### Patients

Patients with iron overload were enrolled from the China Registry of Genetic/Metabolic Liver Diseases (CR-GMLD, Clinical trials. gov: NCT03131427) since October 2014. This study was approved by the Clinical Research Ethics Committee of Beijing Friendship Hospital, Capital Medical University (No. 2016-P2-061-01). Informed and written consent was obtained for the study from all patients.

The inclusion criteria were based on the American Association for the Study of Liver Diseases 2011 practice guidelines on hemochromatosis [6, 11]: (1) elevated ferritin (> 300 ng/mL in men and postmenopausal women or > 200 ng/mL in premenopausal women) and/or transferrin saturation (TS) ≥ 45%; (2) iron overload in the liver and/or spleen on magnetic resonance imaging (MRI) of the liver or liver histology.

The exclusion criteria: (1) alcoholic liver disease, chronic hepatitis B or C, or other chronic liver disease; (2) iron-overloading anemia; (3) parenteral iron overload.

### Clinical and laboratory profiles

The following information were included in the studies: Sex; Age at diagnosis; laboratory data: serum ferritin (SF, a surrogate marker of storage iron), transferrin saturation (TS, the ratio of iron on transferrin); liver chemistry including ALT, AST, GGT, TBIL and ALB; clinical features at presentation: (a) liver fibrosis or cirrhosis, (b) skin pigmentation, (c) arthritis or arthropathy, (d) cardiac involvement (including cardiomyopathy), (e) diabetes or hyperglycemia, (f) hypogonadism.

### Screening for gene variants

Genomic DNA was extracted from whole blood using a Genomic DNA Purification Kit (Qiagen, Valencia, CA). All exons of known HH related genes (*HFE*, *HJV*, *HAMP*, *TFR2* and *SLC40A1*) and exons with mutations in the novel iron homeostasis-related genes (*DENND3* p.L708V and *SUGP2* p.R639Q), were PCR-amplified with their associated boundary regions using primers described in our previous studies [10].

PCR amplification was performed in an ABI Veriti 96 PCR cycler (Applied Biosystems, MA, USA). PCR products were sequenced in forward and reverse orientations using an automated ABI 3730 DNA sequencer (Applied Biosystems). Three predictors, Polyphen-2 (http://genetics.bwh.harvard.edu), SIFT (http://sift.jcvi.org/) and Mutation Taster (http://www.mutationtaster.org/), were used to predict the functional consequence of the identified variants.

We defined pathogenic or likely pathogenic variants as those variants meeting one of the following criteria [12–14]: (1) the variants had previously been reported in the literature; (2) the variants were present in the HGMD, dbSNP, and ClinVar databases; (3) functional effect predictors predicted to be “damaging” by at least two of the three prediction tools were considered to be pathogenic variants; (4) the terms were used by standards and guidelines for the interpretation of sequence variants.

### Statistical analysis

We used SPSS software V.26.0 to conduct all statistical comparisons. Continuous variables were presented as the mean ± standard deviation and compared using one-way ANOVA and LSD-t test, while continuous non-parametric variables were presented as median ± interquartile range and compared using Kruskal–Wallis ANOVA test. Discontinuous variables were compared using chi-square test and Fisher’s exact test. *p* Values of less than 0.05 was considered to be statistically significant.

## Results

### Clinical profiles of the enrolled patients

Thirty-two patients with primary iron overload from the CR-GMLD were recruited to screen for genetic variants in known HH-related genes and novel iron homeostasis-related genes. All the probands with primary iron overload were validated by liver biopsy and/or MRI examinations. Demographic characteristics of patients with non-*HFE* HH are shown in Table 1.

**Table 1:** Demographic characteristics of patients in in different groups with HH

Characteristic	Total (32)	HJV (n = 8)	SLC40A1 (n = 6)	SUGP2 or DENND3 (n = 10)	HH without P or LP variants* (n = 6)	*p*
Male, n (%)	23(71.9)	6 (75)	4 (66.7)	8 (80)	4 (66.7)	0.897
Age, y	45.07 ± 15.94 (18–79)	30.13 ± 12.12	56.33 ± 6.83 ^a^	48.30 ± 16.99 ^a^	48.33 ± 12.55 ^a^	0.007
SF, ng/ml	2631.0 (1115.95, 6371.25)	6153(3246.5,6922.5)	5917.6 (2061.8,9333.8)	972 ^ab^ (670.3,2467.1)	1267 (1033.8,10129.7)	0.010
TS, %	92.30 (81.30,96.10)	94.85 (92.25,97.5)	92.25 (60.43,95.75)	85.0(58.5,96.7)	88.35(70.6,93.5)	0.210
ALT, U/L	72.0 (30.0,104.0)	99.5(67.3,12)	78.5(39.5,141.3)	31(22.5,44.5)	72(18.5,121.5)	0.161
AST, U/L	62.8 (34.6,103.0)	92(64.4,121.0)	60.4(37.6,111.8)	33.4(22.9,48.5)	72(20.7,124.3)	0.051
GGT, U/L	42 (27.00,72.0)	48(34.3,75)	38(19.8,278.3)	29.9(26.5,58.5)	56(22.5,403)	0.622
TBIL, μmol/L	37.88 ± 43.86	17.56 ± 8.85	18.79 ± 10.72	64.14 ± 62.59 ^a^	51.25 ± 48.90	0.094
ALB, g/L	40.30 ± 8.33	43.98 ± 10.12	38.88 ± 3.87	39.14 ± 9.42	37.96 ± 7.58	0.540
Cirrhosis	11	0	5	4	2	0.011
Skin pigmentation	13	4	4	2	3	0.281
Arthropathy	2	1	1	0	0	0.540
Cardiac involvement	5	3	0	0	2	0.042
Diabetes	11	3	5	1	2	0.035
Hypogonadism	9	5	3	1	0	0.020

HH without P or LP variants* = HH without pathogenic or likely pathogenic variants, SF = serum ferritin,

TS = transferrin saturation, ALT = alanine transaminase, AST = aspartate aminotransferase,

GGT = γ-glutamyltransferase, TBIL = total bilirubin, ALB = albumin; Statistically significant differences are denoted as (a) compared to HJV. Statistically significant differences are denoted as (b) compared to SLC40A1

### Gene variants distribution

**Fig. 1:**
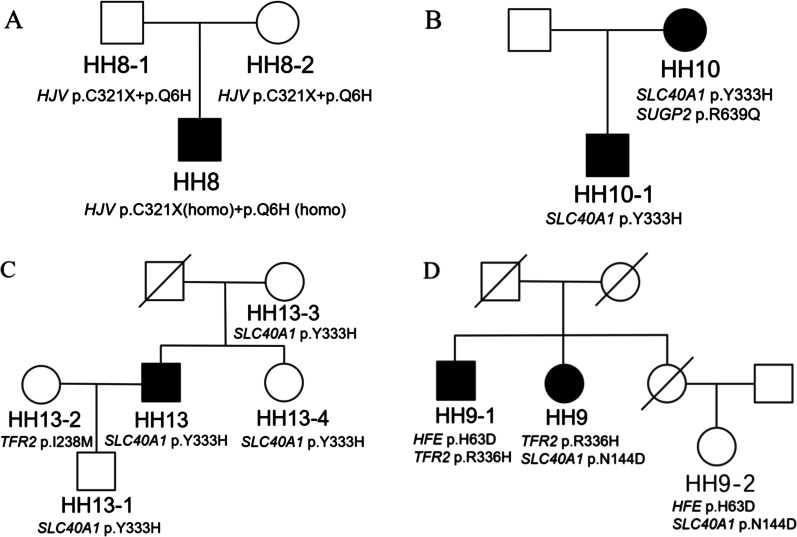
Representative pedigree analysis of four families. **A** Pedigree chart of family HH8. The patient (HH8) harbored homozygous variations of *HJV* p.C321X and *HJV* p.Q6H, which were inherited from their father and mother. **B** Pedigree chart of family HH10. The patient (HH10) harbored compound heterozygous variations of *SLC40A1* p.Y333H and *SUGP2* p.R639Q. **C** Pedigree chart of family HH13. The patient (HH13) harbored a single heterozygous variation of *SLC40A1* p.Y333H, which was inherited from their mother. **D** Pedigree chart of family HH9. The patient (HH9) harbored compound heterozygous variations of *SLC40A1* p.N144D and *TFR2* p.R336H

We found that genetic variants forms of Chinese patients with primary iron overload are mainly non-*HFE*-related combined heterozygous variants. 1 case (3.13%) carried combined heterozygous *HFE* p.C282Y/71X pathogenic variants. 8 cases (25%) carried *HJV* pathogenic variants, among which 4 cases carried homozygous pathogenic variants in *HJV* gene, including p. Q6H, p. F103L, p. Q312X and p. C321X. 7 (21.88%) cases with *SLC40A1* pathogenic variants, 8 (25%) cases with *SUGP2* likely pathogenic variants, 5 (15.63%) cases with *DENND3* likely pathogenic variants. None of damaging or probably damaging variants for *HAMP* and *TFR2* was identified in any cases. Representative predicted pathogenic variants and allele frequency of HH included in this study are shown in Table 2. Representative pedigree analysis of four families are shown in Fig. 1.

**Table 2:** Representative predicted pathogenic variants and allele frequency of HH included in this study

Gene	Location		Nucleotide change	Amino acid alteration		Mutation type	Frequency (this study)	Frequency (Asia/World)	Prediction of Pathogenicity (score)		
									Polyphen-2	SIFT	Mutation Taster
*HJV*	Exon 4		c.963C>A	p.C321X		Nonsense	7/32	5.798e−05/4.062e−06	NA	NA	Disease causing (1)
*HJV*	Exon 4		c.842T>C	p. I281T		Missense	2/32	5.798e−05 /8.121e−06	Probably damaging (1.0)	Damaging (0.000)	Disease causing (0.999)
*HJV*	Exon 4		c.934C>T	p. Q312X		Nonsense	1/32	NA	NA	NA	Disease causing (1)
*HJV*	Exon 3		c.311A>G	p.H104R		Missense	1/32	NA	Probably damaging (0.999)	Damaging (0.000)	Disease causing (1)
*HJV*	Exon 4		c.820G>A	p. V274M		Missense	1/32	0.0012/8.527e−05	Probably damaging (0.997)	Tolerated (0.060)	Disease causing (0.986)
*HJV*	Exon 3		c.309C>G	p. F103L		Missense	1/32	NA	Probably damaging (0.649)	Damaging (0.000)	Disease causing (1)
*SLC40A1*	Exon 5		c.430A>G	p. N144D		Missense	1/32	NA	Probably damaging (1.0)	Tolerable (0.067)	Disease causing (1)
*SLC40A1*	Exon 7		c.997T>C	p. Y333H		Missense	3/32	NA	Probably damaging (1.0)	Damaging (0.022)	Disease causing (1)
*SLC40A1*	Exon 8		c.1531G>A	p. V511I		Missense	1/32	NA	Probably damaging (0.984)	Damaging (0.000)	Disease causing (1)
*SLC40A1*	Exon 5		c.485_487delTTG	p. v162del		Deletion	1/32	NA	–	–	–
*SLC40A1*	Intron	IVS3+10delGTT			–	Splicing	1/32	NA	–	–	–
*SUGP2*	Exon 5		c.1916G>A	p. R639Q		Missense	8/32	0.0388/0.0029	Probably damaging (0.992)	Damaging (0.005)	Polymorphism (0.980)
*DENND3*	Exon 14		c.2122C>G	p. L708V		Missense	5/32	0.0333/0.0029	Probably damaging (0.982)	Damaging (0.003)	Disease causing (1)
*HFE*	Exon 4		c.845G>A	p. C282Y		Missense	1/32	0.0001/0.0332	Probably damaging (1.0)	Damaging (0.000)	Disease causing (1)
*HFE*	Exon 2		c.211C>T	p. R71X		Nonsense	1/32	0.0002/ 1.624e-05	NA	NA	Disease causing (1)

Among these 31 non*-HFE* HH cases, 4 cases harbored homozygous variants, 2 cases harbored homozygous + heterozygous variants, 19 cases harbored heterozygous or combined heterozygous variants, and 6 cases harbored no damaging variants. Genetic characteristics of patients with HH are shown in Table 3.

**Table 3:** Genetic characteristics of 32 patients in HH cases

NO	Age	Gender	SF (ng/ml)	TS (%)	Iron deposition on liver biopsy	Iron overload on MRI	Known HH-related genes	Iron homeostasis -related genes
1	26	M	7004	92.0	Predominant in hepatocytes	Liver	HJV p.Q6H/C321X/I281T	–
2	28	M	6269	95.4	Predominant in hepatocytes	Liver, pancreas	HJV p.Q6H/C321X/I281T	–
3	22	F	2995	89.1	Predominant in hepatocytes	Liver, pancreas	HJV p. Q312X (homo)	–
4	18	M	6678	100	Predominant in hepatocytes	ND	HJV p.Q6H/C321X/H104R	–
5	57	M	4001	93.0	Predominant in hepatocytes	Liver	HJV p.Q6H/C321X/V274M	–
6	36	F	2000	96.0	Predominant in hepatocytes	Liver	HJV p. F103L (homo)	–
7	30	M	11,555	98.0	ND	Liver, spleen, pancreas	HJV p. Q6H(homo)/ C321X (homo)	–
8	24	M	6037	94.3	ND	Liver, pancreas	HJV p. Q6H(homo)/C321X (homo)	–
9	57	F	5886.1	71.1	Hepatocytes and reticuloendothelial cells	Liver, spleen, pancreas	SLC40A1 p. N144D	–
10	66	F	1446.2	92.7	ND	Liver, spleen, pancreas	SLC40A1 p. Y333H	SUGP2 p. R639Q
11	48	M	2267-	91.8	ND	Liver, spleen, pancreas	SLC40A1 p. V511I	–
12	58	M	5949	28.4	Predominant in hepatocytes	Liver, spleen	SLC40A1 p. v162del	–
13	49	M	7445	97.0	Predominant in hepatocytes	Liver, spleen	SLC40A1 p. Y333H	–
14	60	M	15,000	94.0	Predominant in hepatocytes	Liver, spleen, pancreas	SLC40A1 p. Y333H	–
15	79	M	493.8	97.1-	ND	Liver, pancreas	–	SUGP2 p. R639Q
16	63	M	3868	97.7	Predominant in hepatocytes	Liver, spleen	–	SUGP2 p. R639Q
17	67	M	1102	92.3	Predominant in hepatocytes	Liver, spleen	–	DENND3 p. L708V(homo) SUGP2 p. R639Q
18	28	M	738	46.4	Predominant in hepatocytes	ND	–	DENND3 p. L708V
19	38	F	843	96.2	Predominant in hepatocytes	ND	–	DENND3 p. L708V
20	53	F	1402	49.0	Predominant in hepatocytes	Liver, spleen	–	SUGP2 p. R639Q
21	46	M	2000	85.0	ND	Liver, spleen	–	SUGP2 p. R639Q
22	45	M	685	-	Predominant in hepatocytes	ND	–	DENND3 p. L708V
23	33	M	626	68.0	Hepatocytes and reticuloendothelial cells	ND	–	SUGP2 p. R639Q
24	31	M	6000	81.0	ND	Liver	–	DENND3 p. L708V
25	66	F	12,703-	91.7	Predominant in hepatocytes	Liver, spleen, pancreas	–	–
26	50	F	9272	81.6	Predominant in hepatocytes	Liver, spleen, pancreas	–	–
27	53	M	1121	37.6	Predominant in reticuloendothelial cells	Liver, spleen	–	–
28	53	M	773	96.8	Predominant in hepatocytes	Liver	–	–
29	31	M	1220	92.4	ND	Liver, spleen, pancreas	–	–
30	37	M	1316	85.0	ND	Liver, spleen	–	–
31	48	F	7078	99.7	Predominant in hepatocytes	Liver, spleen	SLC40A1 IVS3 + 10delGTT	SUGP2 p. R639Q(homo)
32	28	M	2153	91.7	Predominant in hepatocytes	Liver	HFE p.C282Y/R71X	–

HH = Hereditary hemochromatosis, ND = not done

### Grouping of non-*HFE* HH

Patients with non-*HFE* related HH were divided into four groups in the study, *HJV* HH (Type 2A HH), *SLC40A1* HH (Type 4B HH), *SUGP2* or *DENND3* variants HH and No pathogenic or likely pathogenic variants HH groups, based on the pathogenic variants identified in these cases. Demographic and laboratory characteristics of the four groups of HH cases are shown in Table 1.

Among the 31 cases with non-*HFE* related HH, 2 cases (the first is *SLC40A1* p. Y333H + *SUGP2* p. R639Q, the second is *SLC40A1* IVS3 + 10delGTT + *SUGP2* p. R639Q(homo)) carried two different pathogenic or likely pathogenic variants. The first was grouped into the *SLC40A1* HH due to the definite pathogenicity of *SLC40A1* p. Y333H. Previous functional studies showed that the *SLC40A1* p. Y333H variant was associated with gain-of-function of ferroportin and caused iron overload and organ damage [15]. The second carried two likely pathogenic variants. This patient was a 48-year-old female with SF 7078 ng/ml and TS 99.7%. Liver biopsy suggested that iron deposition was predominant in hepatocytes, MRI suggested iron overload in liver and spleen, and gene test suggested *SLC40A1* IVS3 + 10delGTT + *SUGP2* p. R639Q(homo) combined likely pathogenic variants. We excluded this patient due to difficulty in grouping. Therefore, we finally analyzed the remaining 30 cases.

### Genotype and phenotype associations in different types of non-*HFE* HH

#### HJV HH (Type 2A HH)

There were more males than females in *HJV* HH, the ratio of males and females was 3:1. Mean age at diagnosis of this group of patients was the lowest (30 years) in the four types. Totally, 62.5% of the patients had hypogonadism, half of them developed skin pigmentation, 37.5% had both cardiac involvement and diabetes. Only one case developed arthropathy. None of patients in *HJV* HH developed cirrhosis.

ALT and AST (median 99.5 and 92 U/L) levels increased in this group. GGT (median 48 U/L), TBIL (mean 17.6 μmol/L) and ALB (mean 44.0 g/L) levels were normal. The median SF (6153 ng/ml) and TS (median 95%) levels were highest in four types.

#### SLC40A1 HH (Type 4B HH)

There were more males than females in *SLC40A1* HH, the ratio of males and females was 2:1. Mean age at diagnosis of this group of patients was the highest (56 years) in the four types. Totally, 83.3% of the patients had both cirrhosis and diabetes, 66.6% had skin pigmentation, half of them had hypogonadism. Only one case had arthropathy. None of patients in *SLC40A1* HH developed cardiac diseases.

ALT and AST (median 78.5 and 60.4 U/L) levels increased in this group. GGT (median 38 U/L), TBIL (mean 18.9 μmol/L), and ALB (mean 38.9 g/L) levels were normal. The median SF level was 5918 ng/ml. The median TS was 92%.

#### SUGP2 or DENND3 variants HH

There were more males than females in *SUGP2* or *DENND3* HH, the ratio of males and females was 4:1. Mean age at diagnosis of this group of patients was 48 years. Totally, 40% of the patients had cirrhosis, 20% had skin pigmentation, 10% had both diabetes and hypogonadism. None of patients in *SUGP2* or *DENND3* HH developed arthropathy and cardiac diseases.

ALT (median 31 U/L), AST (median 33.4 U/L), GGT (median 29.9 U/L), and ALB (mean 39.1 g/L) levels were normal in this group. TBIL (mean 64.1 μmol/L) levels were higher than other groups. The median SF level was 972 ng/ml. The median TS level was 85%.

#### HH without pathogenic or likely pathogenic variants

There were more males than females in HH without pathogenic or likely pathogenic variants, the ratio of males and females was 2:1. Mean age at diagnosis of this group of patients was 48 years. Totally, 50% of the patients had skin pigmentation, 33.3% had cirrhosis, 33.3% had both cardiac diseases and diabetes. None of patients developed arthropathy and hypogonadism.

ALT and AST (median 72 and 72 U/L), TBIL (mean 51.3 μmol/L) levels increased in this group. GGT (median 56 U/L) and ALB (mean 38.0 g/L) levels were normal. The median SF level was 1267 ng/ml. The median TS level was 88%.

**Fig. 2:**
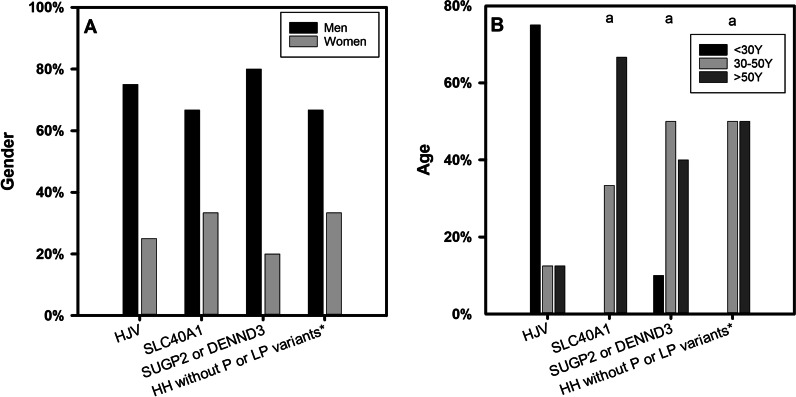
Gender and age at diagnosis of patients with non-*HFE*-related HH. **A** Gender and **B** Age at diagnosis are shown for patients with *HJV* HH, *SLC40A1* HH, *SUGP2* or *DENND3* HH and HH without pathogenic or likely pathogenic variants. Graphs A show the ratios of males and females in the four groups. Variables were compared using chi-square test and Fisher’s exact test. There are no statistical differences between the groups. Graphs B show percentage of patients at different age stages (< 30Y, 30-50Y, > 50Y). Variables were compared using one-way ANOVA and LSD-t test. Statistically significant differences are denoted as (a) compared to *HJV*

**Fig. 3:**
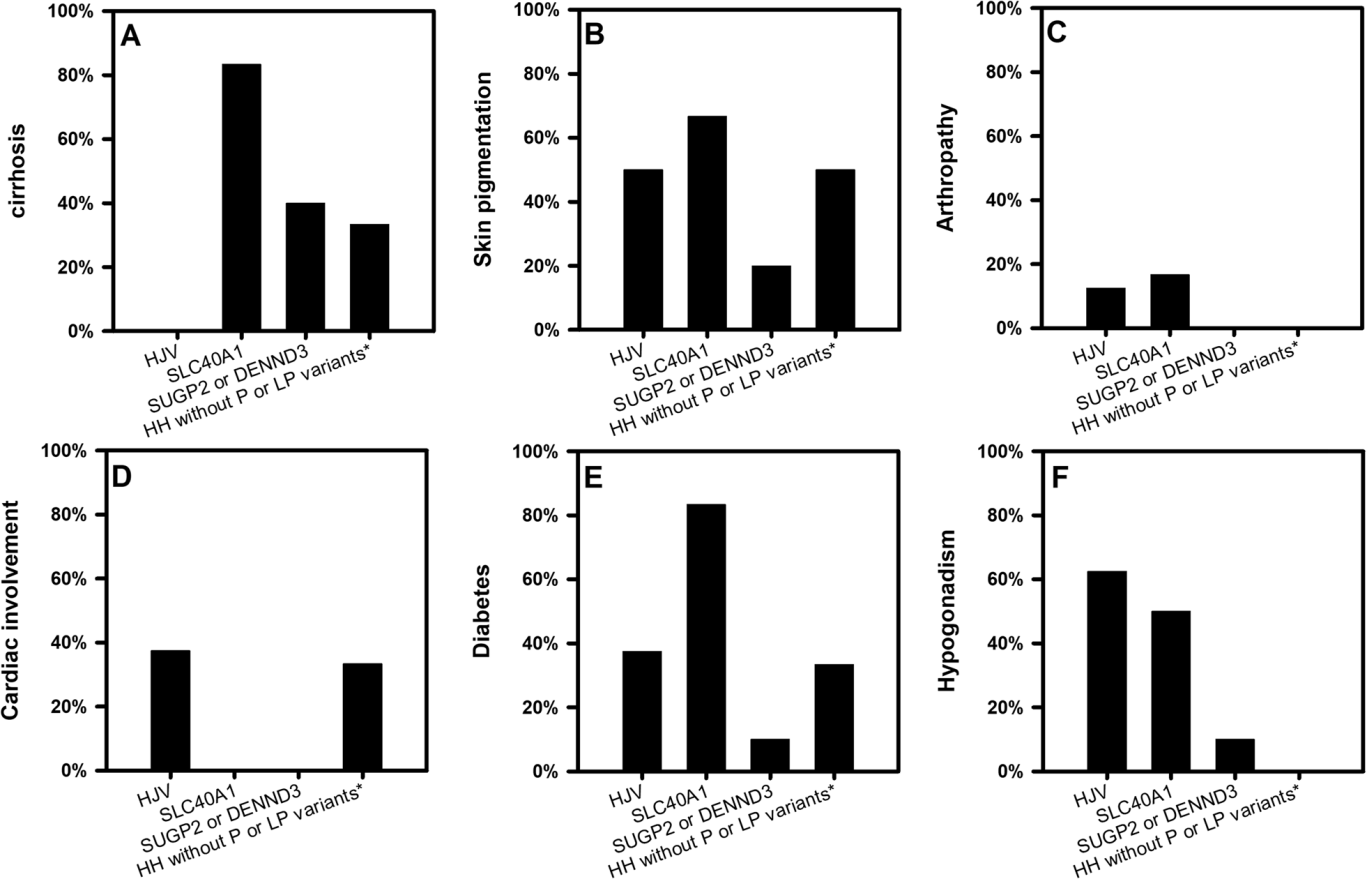
Clinical characteristics at diagnosis of patients with non-HFE-related HH. The presence or absence of clinical characteristics was determined in all patients with genetic diagnosis of *HJV* HH, *SLC40A1* HH, *SUGP2* or *DENND3* HH and HH without pathogenic or likely pathogenic variants. **A** Cirrhosis, **B** Skin pigmentation, **C** Arthropathy, **D** Cardiac involvement, **E** Diabetes and **F** Hypogonadism at diagnosis are shown for subjects in the four groups. Variables were compared using chi-square test and Fisher’s exact test. Differences in clinical characteristics with Cirrhosis, Cardiac involvement, Hypogonadism were statistically significant in the four groups. However, due to the limited sample size, the pairwise comparison showed no significant difference

There were more males than females in all groups (Fig. 2A). The age at diagnosis was statistically different between *HJV* HH and *SLC40A1* HH groups (p = 0.001), between *HJV* HH and *SUGP2* or *DENND3* HH groups (*p* = 0.008), between *HJV* HH and HH without pathogenic or likely pathogenic variants groups (*p* = 0.018), while the comparison between other groups was not statistically significant (Fig. 2B). The incidence of cirrhosis (*p* = 0.011), cardiac involvement (*p* = 0.042), diabetes (*p* = 0.035) and hypogonadism (*p* = 0.020) was statistically significant in the four groups. However, due to the limited sample size, the pairwise comparison showed no significant difference (Fig. 3). HH without P or LP variants* = HH without pathogenic or likely pathogenic variants.

TBIL levels were significantly higher in *SUGP2* or *DENND3* HH when compared to *HJV* HH groups (*p* = 0.032). There was no significant difference about other liver function indices in the four groups. SF levels increased greatly in all the four groups. There were statistical differences between *HJV* HH and *SUGP2* or *DENND3* HH groups (*p* = 0.002), between *SLC40A1* HH and *SUGP2* or *DENND3* HH groups (*p* = 0.01), while the comparison between other groups was not statistically significant. TS increased greatly in all HH groups, with a median TS of 92% in all cases.

## Discussion

The present study demonstrated that the pathogenic gene of HH in China was mainly non-*HFE* genes, which is different from that in Caucasians. We only found one case carried combined heterozygous *HFE* p.C282Y/71X pathogenic variant in our study, which has been reported in our previous study [16]. The remaining 31 cases are non-*HFE*-related HH, especially in *HJV* and *SLC40A1* genes. This suggests that type 2A HH and type 4 HH are major forms of HH in Chinese populations. Also, we analyzed the correlation of the genotype and clinical phenotype of these HH patients.

In the present study, cases with *HJV*-related HH had an earlier age at diagnosis and more severe iron load. In contrast, *SLC40A1* HH, *SUGP2* or *DENND3* HH and HH without pathogenic or likely pathogenic variants occur relatively late, rarely before the age of 30. Furthermore, most of the patients with *HJV* HH had heart failure as the first symptom, whereas there was no cirrhosis in *HJV* HH. A meta-analysis showed that cardiomyopathy is generally seen in individuals with much greater degrees of iron overload [17]. This is in line with the previous report that a much earlier and more serious deposition of iron in the heart in *HJV* HH [18]. However, the exact mechanism for the severer heart disease than the liver disease in *HJV* HH is still not clear.

We also found cases with *SLC40A1* HH had higher prevalence of cirrhosis and diabetes in this studies, which may be related to the severe iron overload and the late onset age of *SLC40A1* HH. Firstly, higher ferritin levels are independently associated with prevalent diabetes [19]. Some studies showed that increased ferritin was associated with increased risk of type 2 diabetes after adjustment for conventional risk factors for diabetes [20]. This relationship between iron and diabetes was also found in gestational diabetes and prediabetes [21, 22]. Secondly, age is known to be a risk factor for diabetes. The older age may increase the more chances of developing cirrhosis and diabetes than *HJV* HH. In addition, severe iron overload in pancreas was observed in some patients, which may be associated with the onset of diabetes.

In our previous study, function study showed that silencing *SUGP2* expression downregulated the level of *HAMP* expression, and a decrease in the level of p-SMAD1/5 and TFR2 was observed in the Huh-7 cell line transfected with the *DENND3* and *DENND3* p. L708V constructs [10]. In the present study, cases with *SUGP2* or *DENND3* HH group showed lower involvement of skin pigmentation, arthropathy, cardiac diseases, diabetes and hypogonadism, when compared to *HJV* HH group; and lower prevalence of cirrhosis when compared to *SLC40A1* HH group. This may be due to the lower accumulation of iron in *SUGP2* or *DENND3* HH group than *HJV* HH group and *SLC40A1* HH group. Therefore, we may infer that the pathogenicity of *SUGP2* or *DENND3* gene variants is weaker than those in *HJV* or *SLC40A1* gene.

It is worth noting that HH without pathogenic or likely pathogenic variations were identified in 6 cases, suggesting that pathogenic variants may exist in other HH-related genes. The second-generation sequencing for those unexplained HH cases would be justified.

Overall, our data showed that there were more men than women among these patients with different non-HFE genotypes. Relevant studies have found that the onset age of female patients is usually later than that of male patients [23]. The prevalence of high serum ferritin levels is higher in males than in females. We think the appearance of symptoms may also be associated to other non-genetic factors, such as alcohol and high-fat diet, and it may explain the protective effect against hemochromatosis by the fact that menstruation delay iron accumulation in women [23]. We found that in the *HJV* HH, *SLC40A1* HH and HH without pathogenic or likely pathogenic variants groups, about half of cases had skin pigmentation, whereas arthropathy occurred in only two cases. This is consistent with the previous reports that skin pigmentation is more prevalent and arthropathy is rare in non-*HFE* HH compared to *HFE* HH [18, 24].

Studies have shown that there is significant positive correlations of SF with TS, ALT and AST [25]. The iron overload of type 2A and type 4 HH was higher than that of the other two groups. From the liver function tests in this study, ALT and AST increased in type 2A and type 4 HH. The rising trend of ALT/AST and SF in this study was consistent with the results of Barton J C [25].

Obviously, this study had some limitations. Firstly, the relatively small number of patients included preclude the conclusive correlation of genotype and phenotype. Secondly, we mainly focused on pathogenic or likely pathogenic variants and did not include the numerous combined heterozygous variants which might also confer various degree of pathogenicity. Thirdly, we did not analyze the histopathological characteristics, due to lack of liver biopsy in some patients.

## Conclusions

In conclusion, this study suggested variants in non-*HFE* genes were the main pathogenic genes in Chinese HH patients. Cases with *HJV*-related HH had an earlier age at diagnosis and the more severe iron load, whereas more cases with *SLC40A1* HH had cirrhosis and diabetes. *SUGP2* and *DENND3* were likely pathogenic variants for HH in China.

## Data Availability

The datasets used and/or analyzed during the current study are available from the corresponding author on reasonable request.

## Abbreviations

HH: Hereditary hemochromatosis
*HFE*: Haemochromatosis
*HJV*: Hemojuvelin
*HAMP*: Hepcidin
*TFR2*: Transferrin receptor 2
*SLC40A1*: Ferroportin
*DENND3*: DENN domain-containing protein 3
*SUGP2*: SURP and G patch domain containing 2
MRI: Magnetic resonance imaging
SF: Serum ferritin
TS: Transferrin saturation
ALT: Alanine transaminase
AST: Aspartate aminotransferase
GGT: γ-Glutamyltransferase
TBIL: Total bilirubin
ALB: Albumin
PCR: Polymerase chain reaction

## References

[1] Pietrangelo A (2010). Hereditary hemochromatosis: pathogenesis, diagnosis, and treatment. Gastroenterology.

[2] Crownover BK, Covey CJ (2013). Hereditary hemochromatosis. Am Fam Physician.

[3] Pericleous M, Kelly C (2017). The clinical management of hereditary haemochromatosis. Front Gastroenterol.

[4] Adams PC, Speechley M, Kertesz AE (1991). Long-term survival analysis in hereditary hemochromatosis. Gastroenterology.

[5] Brissot P, Pietrangelo A, Adams PC, de Graaff B, McLaren CE, Loréal O (2018). Haemochromatosis. Nat Rev Dis Primers.

[6] Bacon BR, Adams PC, Kowdley KV, Powell LW, Tavill AS; American Association for the Study of Liver Diseases. Diagnosis and Management of Hemochromatosis: 2011 Practice Guideline by the American Association for the Study of Liver Diseases. Hepatology. 2011;54(1): 328–343. 10.1002/hep.24330 PMC3149125 21452290

[7] Pietrangelo A (2004). Hereditary hemochromatosis-a new look at an old disease. N Engl J Med.

[8] McDonald CJ, Wallace DF, Crawford DH, Subramaniam VN (2013). Iron storage disease in Asia-Pacific populations: the importance of non-HFE mutations. J Gastroenterol Hepatol.

[9] Wang Y, Du Y, Liu G, Guo S, Hou B, Jiang X (2017). Identification of novel mutations in HFE, HFE2, TfR2, and SLC40A1 genes in Chinese patients affected by hereditary hemochromatosis. Int J Hematol.

[10] Lv T, Zhang W, Xu A, Li Y, Zhou D, Zhang B (2018). Non-HFE mutations in haemochromatosis in China: combination of heterozygous mutations involving HJV signal peptide variants. J Med Genet.

[11] Adams P, Altes A, Brissot P, Butzeck B, Cabantchik I, Cançado R (2018). Therapeutic recommendations in HFE hemochromatosis for p.Cys282Tyr (C282Y/C282Y) homozygous genotype. Hepatol Int.

[12] Dong C, Yu B (2011). Mutation surveyor: an in silico tool for sequencing analysis. Methods Mol Biol.

[13] Minton JA, Flanagan SE, Ellard S (2011). Mutation surveyor: software for DNA sequence analysis. Methods Mol Biol.

[14] Richards S, Aziz N, Bale S, Bick D, Das S, Gastier-Foster J (2015). Standards and Guidelines for the Interpretation of Sequence Variants: A Joint Consensus Recommendation of the American College of Medical Genetics and Genomics and the Association for Molecular Pathology. Gene Med.

[15] Zhang W, Xu A, Li Y, Zhao S, Zhou D, Wu L (2019). A novel SLC40A1 p.Y333H mutation with gain of function of ferroportin: a recurrent cause of haemochromatosis in China. Liver Int.

[16] Zhang W, Wang X, Duan W, Xu A, Zhao X, Huang J (2020). HFE-Related Hemochromatosis in a Chinese Patient: The First Reported Case. Front Genet.

[17] Neghina AM, Anghel A (2011). Hemochromatosis genotypes and risk of iron overload-a meta-analysis. Ann Epidemiol.

[18] Sandhu K, Flintoff K, Chatfield MD, Dixon JL, Ramm LE, Ramm GA (2018). Phenotypic analysis of hemochromatosis subtypes reveals variations in severity of iron overload and clinical disease. Blood.

[19] Yeap BB, Divitini ML, Gunton JE, Olynyk JK, Beilby JP, McQuillan B (2015). Higher ferritin levels, but not serum iron or transferrin saturation, are associated with Type 2 diabetes mellitus in adult men and women free of genetic haemochromatosis. Clin Endocrinol (Oxf).

[20] Creighton Mitchell T, McClain DA (2014). Diabetes and Hemochromatosis. Curr Diab Rep.

[21] Afkhami-Ardekani M, Rashidi M (2009). Iron status in women with and without gestational diabetes mellitus. J Diabetes Compl.

[22] Sharifi F, Nasab NM, Zadeh HJ (2008). Elevated serum ferritin concentrations in prediabetic subjects. Diab Vasc Dis Res.

[23] Allen KJ, Gurrin LC, Constantine CC, Osborne NJ, Delatycki MB, Nicoll AJ (2008). Iron-overload-related disease in HFE hereditary hemochromatosis. N Engl J Med.

[24] Husar-Memmer E, Stadlmayr A, Datz C, Zwerina J (2014). HFE-related hemochromatosis: an update for the rheumatologist. Curr Rheumatol Rep.

[25] Barton JC, Barton JC, Adams PC (2017). Clinical and Laboratory Associations with Persistent Hyperferritinemia in 373 Black Hemochromatosis and Iron Overload Screening Study Participants. Ann Hepatol.

